# Tacit knowledge in water management: a case study of Sponge City

**DOI:** 10.14324/111.444/ucloe.000031

**Published:** 2022-02-01

**Authors:** Zeyu Yao, Sarah Bell

**Affiliations:** 1 Institute for Environmental Design and Engineering, University College London (UCL), Gower Street, London WS1E 6BT, UK; 2 Melbourne Centre for Cities, University of Melbourne, Grattan Street, Parkville, Victoria 3010, Australia

**Keywords:** Sponge City, tacit knowledge, social capital, China, integrated urban water management, knowledge transfer, urban planning

## Abstract

Sustainable, resilient urban water management is fundamental to good environmental and public health. As an interdisciplinary task, it faces enormous challenges from project complexity, network dynamics and the tacit nature of knowledge being communicated between actors involved in design, decisions and delivery. Among others, some critical and persistent challenges to the implementation of sustainable urban water management include the lack of knowledge and expertise, lack of effective communication and collaboration, and lack of shared understanding and context. Using the Chinese Sponge City programme as a case study, this paper draws on the perspectives of Polanyi and Collins to investigate the extent to which knowledge can be used and exchanged between actors. Using Collins’ conceptualisation of the terrain of tacit knowledge, the study identifies the use of relational, somatic and collective tacit knowledge (CTK) in the Sponge City pilot project. Structured interviews with 38 people working on a Sponge City pilot project provided data that was rigorously analysed using qualitative thematic analysis. The paper is original in identifying different types of tacit knowledge in urban water management, and the potential pathways for information and messages being communicated between actors. The methods and results provide the groundwork for analysing the access and mobilisation of tacit knowledge in the Sponge City pilot project, with relevance for other complex, interdisciplinary environmental projects and programmes.

## Introduction

Sustainable urban water management aims to deliver safe, reliable and secure water and sanitation services, by mobilising stakeholders to ensure good public health and improve social, economic and ecological outcomes [1–3]. Sustainable approaches encourage integrated management of water supply, stormwater and wastewater infrastructure, and stronger integration of water with urban design and decision making. Working in urban water management demands the ability to work with professionals from other disciplines, including the ability to communicate complex ideas with people who do not share background knowledge [4]. More than a decade ago, Brown et al. [5] found that planning for stormwater management in Australian local government was dominated by engineering consultants, and project implementers did not have the skills, expertise or mentality required to work with non-technical communities. More recently, Cosgrove and Loucks [4] commented that stakeholder engagement and interdisciplinary working are yet to be achieved in urban water management, calling for water managers be involved in the earliest stages of urban planning to enable productive interaction with other sectors.

Sustainable approaches to urban water management are increasingly relevant as cities around the world address challenges of climate adaptation, resilience and growing populations [1], leading to ever higher recognition of the importance of inter-disciplinary interaction and coordination [6]. In China, Sponge City is a flagship initiative implemented by the central government to promote a more sustainable and healthier environment, focusing on urban stormwater treatment and control [7,8]. It has a set of focused objectives targeting water management in urban areas. The word ‘sponge’ was initially used by researchers to refer metaphorically to the flood management abilities of the natural environment [9]. The city as a ‘sponge’ is adaptive and resilient to changes in the environment, especially during high rainfall events [7]. The Sponge City is resilient to flooding by optimising the design of urban landscapes to capture and disperse water, including increased use of green infrastructure measures such as rain gardens, green spaces and wetlands. The foundation of the Sponge City concept is similar to international models such as low impact development (LID) and best management practices (BMP) in the United States, sustainable urban drainage (SUDS) in the United Kingdom and water sensitive urban design (WSUD) in Australia [1].

Cross-disciplinary and cross-sectoral collaboration can lead to exchanges of knowledge and expertise, and the realisation of such requires effective communication and understanding. In the context of urban water management, knowledge transfer is exceedingly challenging because actors come together with different worldviews and languages. Sometimes, such knowledge possessed by each actor is difficult to explain and is difficult for an unfamiliar person to understand. The integrated management of urban water relies on a collective network of such tacit knowledge, and this study focuses on China’s Sponge City initiative to investigate the factors that influence the access and mobilisation of the knowledge resources.

Since it began in 2014, the Sponge City initiative has faced a variety of challenges in design and planning, implementation, construction, management and evaluation. Pilot cities were chosen to represent a wide range of social, economic and environmental statuses, and it was anticipated that each city would approach the projects with different perspectives and expectations [10]. However, universal challenges have been experienced in most pilot cities resulting from knowledge gaps across different sectors and disciplines, which may lead to misinterpretation of objectives and requirements, over-simplification or complication of problems and solutions, as well as implementation gaps [11].

Tacit knowledge is difficult or impossible to communicate on paper, and is often neglected in interdisciplinary, cross-sectoral project planning and implementation [12]. Knowledge is kept tacit due to the barriers that arise because of differences in perspectives, responsibilities and interests [13], differences in capacities, capabilities and thought and learning processes [14,15], as well as physical constraints such as lack of time or facilities. Acknowledging debates surrounding definitions of tacit knowledge and the mismatch of epistemic and tacit concepts of knowledge [16,17], this study adopts a pragmatic stance to tackle the problematic situation where some forms of knowledge are more difficult to communicate and comprehend than others.

This paper distinguishes the types of tacit knowledge, identifies and categorises them in the context of a Sponge City pilot. It is original in its focus on the importance of tacit knowledge gaps between professionals with different backgrounds and experiences. The paper begins with an overview of the definitions and theories of tacit knowledge, and its relevance to urban water management. This is followed by a description of Sponge City, outlining the main aims and rationale of the initiative as well as the obstacles in interdisciplinary and inter-sectoral communication and learning. Next, the rigorous qualitative research design and data analysis methods are defined. The next section discusses the findings and provides some conclusions and recommendations for future work.

## Tacit knowledge and sustainable urban water management

Urban water systems comprise a variety of components, from the treatment of water for different purposes to the draining of rainfall runoff from urban streets. The difficulty of water management not only resides in the structural and technical complexities but urban social dynamics create layers of complications and uncertainties. It is a complex system made up by multiple disciplines and professions, each occupying a niche environment and commanding niche knowledge that is difficult to communicate to each other.

Many studies have identified challenges of multi-disciplinary working in urban water management. Challenges relate to policy, resources, governance, and individual and societal perceptions, attitude and behaviours [4,18–22]. This study zoomed in on a root cause of the abovementioned challenges – the difficulties regarding the communication of knowledge between actors. More specifically, tacit knowledge is especially difficult to communicate with others, as it is embedded in each person’s perspective and experience and is mobilised by interactions within social networks.

The concept of tacit knowledge began with Polanyi as he placed scientific knowledge and rational thinking back into the environmental, social, cultural and personal context surrounding the knowledge. Polanyi argued in the book *The Tacit Dimension* [23] that all knowledge relies on personal judgement, and more specifically that ‘we can know more than we can tell’. While he invigorated the discussion on the role of personal experiences in the sharing of scientific knowledge in the well-known book *Personal Knowledge Towards a Post-Critical Philosophy* [24], the theoretical framework of this research is inspired mainly by his theoretical positions outlined in *The Tacit Dimension*. Polanyi spoke of ‘knowing’ – the ‘knowing what’ and the ‘knowing how’, to cover both theoretical and practical knowledge [23]. In Polanyi’s [23] original conceptualisation of tacit knowledge it is deeply personal, held within the body and unable to be made explicit. Two characteristics of knowledge are highlighted by Polanyi: that it relies on personal judgment, and we can know more than we can tell. In his articulation of the concept, all knowledge is either tacit or rooted in tacit knowledge, ruling out the possibility of drawing a clear boundary between ‘tacit’ and ‘explicit’ knowledge.

Collins’ terrain of tacit knowledge provides a broader framework of analysis, expanded from Polanyi’s conceptualisation of tacit knowledge [25,26]. While Collins also conceptualises ‘tacitness’ as a continuous spectrum from ‘weak’ to ‘strong’, he believed that the use of the term ‘tacit knowledge’ has been too broad and imprecise. As the goal of understanding tacit and its explication is to enable its communication, he likens the act of communication to jumping across a gap between two buildings [25]. Collins argued that although all tacit knowledge is obtained by humans through interacting in society, there are different ways to acquire it as the length of ‘gap between the two buildings’ varies.

The three levels of tacit knowledge introduced by Collins are relational, somatic and collective. Collins treated tacit knowledge as more than just a concept but realistically. To jump across the gaps of various lengths, the communication of different types of tacit knowledge should meet different conditions. Relational tacit knowledge (RTK) is mechanically able to be explicated because it refers to knowledge that is intentionally or unintentionally unrevealed. These pieces of knowledge are on the borderline of explicit and tacit. They are categorised as ‘tacit’ knowledge by Collins because they can be but are not made explicit for various reasons. An example of RTK is when a modeller does not realise that a designer fails to understand the jargon being used. As explained here, RTK usually can be communicated if the knowledge is revealed ‘better’ or elaborated further. In other words, it is the piece of knowledge that needs to be modified in order to enable communication.

Somatic tacit knowledge (STK) refers to knowledge that can potentially be broken down into steps to a point where a machine can replicate the performance, but one does not follow each detailed step to use knowledge to perform a task. This is also the type of tacit knowledge that fits Polanyi’s description of tacit the best, where he described that while one can formalise the rules for balancing a bicycle, one does not apply these rules when acting out the riding of a bicycle. In contrast to RTK, the communication of this type of tacit knowledge requires both the modification of the piece of information and the physical change of the receiver of the information.

Collective tacit knowledge (CTK), on the other hand, is unlikely to be made explicit because it is located in human collectivities, so the only way to acquire it is to be embedded in a specific society [26]. While Polanyi’s example of riding a bicycle has become a typical example of tacit knowledge, Collins is positioned to focus on the collective or society level instead of individual or body level of tacit knowledge [25].

## China’s Sponge City programme

Sponge City is a national programme in China, which is a joint initiative of the Ministry of Finance, the Ministry of Housing and Urban-Rural Development and the Ministry of Water Resources. Initially targeting urban stormwater treatment and waterlogging control, it evolved to be a national strategy to achieve a ‘new-type of urbanisation’ and ‘overall construction of a well-off society’ for China [27]. The first batch of 16 ‘pilot cities’ were selected in 2015, and a further batch of 14 ‘pilot cities’ were selected in 2016. Like other programmes in integrated urban water management, Sponge City encourages the practice of the ‘naturalisation’ of the urban water cycle, diversification and decentralisation of water sources and infrastructure, consideration of water conservation and resource efficiency, and integration of water sub-sectors within cities and beyond, as well as engagement with other sectors and communities [7,13,28,29].

Water management in Chinese cities reflects large regional differences in climate and historical practices [30–32]. Compared to the southern Yangtze River basin, where water resources are abundant and stable, the north of China experiences a high seasonal variation of precipitation and much lower per capita water availability [33]. By comparing the water supply and management practices of Beijing and Shanghai, Cosier and Shen [30] found that the environmental and economic contexts account for the differences in their approaches towards the implementation of national policies and regulations. Specifically, northern cities such as Beijing tend to have a better implementation of planned water use and water-saving systems, while southern cities that are more abundant in water tend to be better at adopting water supply and use contracts because they have fewer incentives to restrict water consumption.

Integrated urban water management in China has been interpreted as the unification of all water-related administrative units under a single ministry, rather than the holistic management of the urban water cycle in the coordination of the hydrological cycle with inter-disciplinary and inter-sectoral efforts. To achieve stronger integration of urban water management using the Sponge City concept, there needs to be an alignment of interests, objectives and knowledge capacity at all levels. It is not the lack of willingness to introduce the Sponge City concepts into local government planning agenda that warrants concerns, but rather is the potential lack of knowledge and support needed by the local authorities to adhere to the water management principles as outlined in the national policy.

While many disciplines that are not traditionally involved in urban water management or drainage design are more present in Sponge City projects, actors are facing the challenge of collaborating with disciplines that they never had to work with before. The Sponge City programme requires a higher level of trans-jurisdictional collaboration and coordination, as well as a higher level of inter-disciplinary and inter-sectoral communication and learning. Therefore, it is necessary to understand the factors that influence how Sponge City actors, with different worldviews and technical language, acquire and exchange tacit knowledge.

## Methods

The Sponge City initiative consists of 30 national-level pilot cities and thousands of participants from a range of professions. The initiative began in 2015, with the first batch of cities completing in 2018, and the second batch in 2019. Many of the participants are simultaneously involved in multiple pilot cities, so it is appropriate to adopt the case study method to seek extensive information from a small sample that is representative of the actors involved in Sponge City pilot projects. A series of semi-structured interviews was conducted over 3 months with 38 people, to measure ‘how knowledge is being shared’ in a Sponge City pilot city. The interviews explored various levels and types of knowledge possessed and used by the actors, the mechanism and the quality of their interactions during the projects. A convenience sampling method was used to identify the initial interviewees, although a strict boundary was drawn where only people who worked on the Sponge City pilot projects (2014 to date) were considered. This group of actors included the contacts established during the initial field trip as well as researchers in universities that became acquainted through personal connections. During the interviews, the actors were asked to identify other actors with whom they interacted and exchanged information. The participants were not asked to recall the names of individual actors. Instead, they identified the organisations or groups of individuals. Interviews were transcribed and analysed to build a network of connections for each participant and to identify key themes in how participants described knowledge communication and learning within the Sponge City programme.

The actors interviewed can be divided into four groups based on their professions, as determined by the type of organisation in which their work is based. The groups are university (U), government (G), private sector (C), professional organisations (P). The distinction between companies and professional organisations is made by whether an organisation is affiliated with a government bureau or commission; if not, then it falls under the ‘private sector’ category. Many actors work under different titles and the group that the actor belongs to derives from his or her main title.

Using NVivo 11 Pro software, an initial round of coding was conducted in the original language to preserve meaning and avoid misinterpretation. This round of coding was deductive, where pre-determined codes were derived from the Sponge City guideline [7] and the interview questions. Hereafter, an inductive coding process was carried out as themes emerged from the interviews; at this stage, many codes were collapsed into more condense categories. Then, another deductive process of coding was completed, using more meaningful units of analysis derived from Collin’s conceptualisation of tacit knowledge.

## Results and discussion

The Sponge City projects in general follow three major stages [7], following the first stage where responsibilities have been distributed from the municipal government to the various government agencies, the project proceeds to the ‘planning’ stage where policies regarding technical guidance, implementation and evaluation (reward) are developed. The final stage is where the design, construction and operation and maintenance of each specific project takes place. At each stage, the tasks involve all three components of tacit knowledge, which require some from RTK, some from STK and some from CTK.

From relational to collective, the conditions that need to be changed in order for the knowledge to be made explicit become more complex and difficult to satisfy. The stronger the tacit knowledge, the more difficult it is to ‘eliminate the contingencies of human relationships, history, tradition and logistics’ [25] (p.98). Using the terrain of tacit knowledge conceptualisation, this section demonstrates various types of tacit knowledge that emerged from the interviews with the Sponge City actors and discusses the possible causes for knowledge to become or remain tacit.

### Collective tacit knowledge

There are certain types of knowledge contingent upon factors or conditions existing in the collective society. Collins [25] pointed out that ‘the individual is a temporary and leaky repository of collective knowledge’ (p.133). What causes CTK to be harder to communicate is that the recipient entity must be flexible to change according to changes in the circumstances and the societies where they are located [25]. While Collins described driving in China as a form of CTK because one not only has to know how to operate a vehicle but also know the written and unwritten rules one should abide by on the roads in China. In the case of the Sponge City projects, the interviews revealed three different contexts of CTK.

#### Adapting international practices in the context of Sponge City

Firstly, any adaptation and comparison between Sponge City and other international sustainable urban water practices should consider the social circumstances and society’s unspoken rules. Meanwhile, the societal rules and Chinese water culture that governs the relationships between water professionals are evolving with time as well. In his book *The water kingdom*, Philip Ball drew attention to the historic anthropocentric view of the environment and nature in China. From the ancient Confucian’s idealism to the recent history of dam building, Ball claims that the culture itself is not conducive to environmental protection and preservation, whilst also holding that ‘none of this suggests that there is something uniquely bad for the environment about an authoritarian, socialist form of government’ [34] (p.296). In more recent times since 2012, the engineering intensive projects have started to shift towards the development of ‘ecological civilisation’. Actors from different fields brought up the difficulties in adopting and adapting the Sponge City concepts and relevant methodologies to the needs of China. There were 30 references from 21 interviews pointing to the importance of the context of the knowledge, and 21 references from 10 interviews referred to cultural differences as a source of challenges. As the concepts that eventually became Sponge City have a foreign origin, many actors raised the concerns of applying their existing knowledge to a relatively foreign framework that is not thoroughly adapted to the Chinese context:

*Chinese is a language full of ambiguities. “Sponge city” actually means low impact development, and that’s what it should be called. The problem is that there is not a methodology for China, I personally think the ‘sponge city’ methods is not suitable for China*. **(U5)**

*I think the most difficult in China is that it’s not clear who is pushing/leading the project, whether there are clear instructions that can tell me what to do*? **(C22)**

#### Different practices and cultures across the cities in China

One of the unique characteristics is the top-down information asymmetry, and there were 69 references from 25 out of 38 interviews referred to this point. Many actors pointed out the top-down characteristics of the network and its benefits. The description ‘top-down’ is mentioned frequently in the interviews. At first glance, environmental governance in China seems to be top-down, command and control or authoritarian, where national regulation dominates and the policy process tends to be non-participatory [35,36]. A closer look at the national and local government relations can reveal that policy implementation in China is more complicated than previously anticipated by both researchers and the national government. China’s water resources management adopts policies and laws that are centrally set and locally administered, where a multi-level jurisdictional framework (national, provincial, prefecture and county levels of administration) is combined with a catchment-based approach to river basin management [30]. This is a very complicated situation for water management. While all agencies at a central level have corresponding line agencies at the provincial, prefecture and county levels, who in turn look to the national agencies for technical guidance and implementation of laws, these agencies report administratively to the local governments [33]. The Chinese administrative structure can be characterised by ‘a line’ (tiao) and ‘a block’ (kuai). The ‘line’ refers to hierarchical relations from the Communist Party of China (CPC) committee down to the provincial government, while the ‘block’ refers to the government network at the provincial and city level. The ‘block’ exists because the local governments have considerable autonomy over their own policies. Therefore, it is not guaranteed that the intent of the central government can be met with a similar level of implementation support from the local government. It creates another layer of complexity for integrated management because each party involved may have different or even conflicting priorities and objectives.

*Ningbo has a good foundation, and the local economic condition has been quite good, and the local government officials and officers are very effective and efficient…Compared to Ningbo, [Puyang] has a lower economic status, and the local committees and bureaus have unreliable understanding of Sponge City, not sure what the concepts are referring to and what the objectives are*. **(U2)**

Moreover, cities in China have inherited their own water culture and remnants of physical structures of historical waterworks, and they influence their modern-day practices to different extents. As a city chosen for the Sponge City pilot programme, Ningbo has a long history with what can be described as ‘blue-green’ water management. Early in the city’s history, water control works were constructed using natural materials such as stone, mud and soil, where plants used for reinforcement were able to stabilise the soil and provide food for silkworms [32]. At the time, the system of small waterways and waterbodies supported biodiversity while meeting the society’s needs for irrigation, freshwater and drainage, but urbanisation and industrial development caused a shift from ‘natural’ to grey infrastructure, and the surface water network functions primarily as urban drainage and flood control as agricultural lands are converted to impervious surfaces [32]. In recent times, Ningbo has been actively pursuing sustainable urban water management, even before Sponge City projects started. Similarly, the city of Shenzhen has also been experimenting with concepts similar to Sponge City for a while:

*There is also a reason why Ningbo is taking part in Sponge City, and a key reason is that we have a good foundation. We have done some exploration [in this area] with Australia and collaborated with the US on building ecological barriers before. Both were similar to Sponge City…also considering natural [solutions]. Ningbo is a JiangNan Water Town, so its development historical has always been tied to its water*. **(G1)**

*It’s better in Shenzhen, since [they have been doing it] since fairly early on, a professor from the US has been working on LID in Guangzhou and Shenzhen for a long time. Shenzhen also has done some data collection from experiments, and the Shenzhen urban planning institute has very rich experience and history…The [Sponge city] guideline of Shenzhen is good, it specifically targets the city of Shenzhen*. **(C19)**

*Differences between disciplines and fields*: the divide between certain different disciplines and fields the actors work in can be akin to that between different cultures. For example, the same mathematical equations or the same set of data can be used, but their application and implications may be very different, thus causing a chasm of understanding and priorities between the disciplines.

*[T]here is a battle between the disciplines involved in Sponge City. Landscape architects think that they should be leading the projects, but municipal engineers believe that landscape architects aren’t knowledgeable enough*. **(C19)**

*Regarding the planning making, I think each [planning] company/committee has its own characteristics and specialties. What we do is very different from other planners, we are very different in our understanding and interpretations of Sponge City*. **(P2)**

### Somatic tacit knowledge

STK is knowledge that is stored and embodied by the human body and brain; therefore, it is limited by the capacity of the human body, which means it could be more difficult to eliminate the contingencies; however, it is not necessarily more difficult to communicate compared to RTK [25]. The communication of STK requires not only a modification of the information, but also the physical transformation in the recipient, be it in the brain or the body. The bike riding example Polanyi [23] described is an example of STK, because this is a type of knowledge that even though it can be explicated it needs to be carried out intuitively by a human, and the information is stored in the brain and muscles.

The following examples from several Sponge City project actors illustrate how changes in the receiving entities affect the success of knowledge communication. The scenarios described by actors C5 and C20 are examples of communication being hindered because the actors rely on their experience and intuition, and they are not able to explain them fully to another actor. Meanwhile we can see that the knowledge possessed by the actors are not easily captured formally and performed mechanically:

*Regarding the construction of the rainwater catchment basin/well, the design drawing dictates 5 cm, [but I built] a little more than the drawing, since there were some special circumstances […] the construction supervisor said the height is not right, so I said…if you must build strictly according to the drawing, you’ll find there are several places that don’t match the drawing. If you check it like that than we can’t do our job anymore*. **(C5)**

*It’s been only 2–3 years since Sponge City started so it’s not a mature system yet, and there are no stable written guidance and rules, so during the design phase there are many disagreements between the experts and between the experts and other actors. Whether to use more green or grey infrastructure in a community project depends on the past design experience of the designer and the local conditions*. **(C20)**

Actors tend to find communication to be smoother when the governmental actors have either been introduced to the language in past projects or are becoming familiar with the use of codified information in the form of guidelines and standards, as well as by learning during their current projects. The following quote is an example of successful explication of STK by explanation:

*[The local government actors] have improved a lot, for example, when we are discussing the community design, one of them would express how the goals are set too high, but another would rebut that it’s attainable since the community has a high percentage of vegetation cover. So, some of them would understand how certain types of community can have higher or lower goals. They are aware of that, but it wasn’t like that when we started. We used to spend a long time on explaining the total runoff control, what it is, how it can be attained*. **(C7)**

### Relational tacit knowledge

RTK refers to knowledge that is intentionally hidden, is not revealed due to contingencies of time and place or is unintentionally concealed without realising other people does not understand it. This is the type of tacit knowledge that emerged most frequently from the interviews. Collins [25] characterises this type of tacit knowledge as ‘weak’ tacit knowledge, and its communication requires some form of transformation of the piece of information being transferred. On the other hand, the senders and receivers of RTK share enough cultural similarity and what is notable is how they relate to each other due to personal inclinations or those acquired from their social groups [25].

Concealed knowledge, just as indicated by the term, is kept hidden or a ‘secret’, and thus it is not transferred from one person to another. This type of knowledge sits on ‘the borderline of the explicit and the tacit’ [25] (p.93), and the conversation from tacit to explicit can happen by including the sender and the receiver of information in the same conversation or located in the same framework of time and space. Urban planner C17 and civil engineer P1 revealed in their interviews that difficulties of identifying with another person or group of people prevented them from being more open to cooperation and learning, and from developing trust in another person or group of actors. Also, the level of communication is high between actors from the same unit of the same organisation:

*It’s different than [projects in the past that] people are not sure which group to identify with, because it’s not clear anymore what each discipline or profession is in charge of anymore*. **(C17)**

*The problem now is that people are not sure whom they are working for*. **(P1)**

Many of the actors interviewed found the act of learning and communicating hindered the quality of their exchanges. What also made knowledge transfer and learning difficult, is the inability of actors to adequately explain a concept to another individual. This is an example of the challenges in communicating ostensive knowledge, where the actors must go beyond using a description in words and involve connections through other senses, such as the showing of an artefact [25]:

*Also, we would go to the construction site to research and study…We can’t be all talks, we also have to go to the sites to see how it’s done*. **(C14)**

Even when the actors are not intentionally or unintentionally hiding any ‘secret’ knowledge, sometimes the task could be too logistically demanding for actors to carry out. Actor U6 explained that a good way to collaborate is to let each group or unit carry out their own tasks and come together:

*By working together, I mean we each carry out our own tasks separately*. **(U6)**

In these cases, the actors expected the collaborators to demonstrate competent skills within their own expertise, and such trust dwindles for other types of tasks. However, while this type of relationship enables a better collaboration process, this type of manifestation of trust limits the exchange of knowledge and combination between actors from different disciplines, as the actors did not engage in social exchange or learn beyond the already codified knowledge.

There was also knowledge being kept tacit due to mismatched salience and unrecognised knowledge. If the knowledge is not concealed, too complex to be described or logistically demanding, the transfer of knowledge may be contingent upon a person recognising the values of knowledge they possess, or the knowledge possessed by another person. C12 is an urban planner but working on Sponge City plans required her to have some understanding of topics such as hydrology and urban flooding. Despite working on the same project in the same office, she was not able to learn as much as she would have liked to from her colleagues who were the experts on water topics. Previously, it is shown that time and effort are required to assimilate and exploit new knowledge, the interviews revealed that the task of explaining becomes more complicated when there is not enough overlapping of knowledge between the actors. Designer C19 had similar experiences in another company. As a landscape designer who has had experience working on water models before, she struggled to explain certain concepts and indices used in the models to other designers who did not have prior knowledge of water modelling:

*Currently it’s quite frustrating. I know a little about Sponge City and I have done some modelling before, but I had a harder time when I needed to discuss with my colleagues. Communication with them was difficult, because although they understand the national standards and common concepts such as volume rate, greening rate, but it’s more difficult for them to think back to the concepts such as annual total runoff control rate and low elevation green belt rate*. **(C19)**

While the previous cases demonstrate knowledge being concealed due to mismatched saliences between actors, knowledge can also be concealed because neither of the actors recognise the value of what they know. U10 expressed the difficulties in communicating between designers and material suppliers due to the fact that neither of them realised the differences in the local sand composition and the challenges this may cause:

*[T]he most important actor is the material supplier, since the more information they have regarding the materials they have, the better they are equipped to know what to use in the designs. [Last year], we were looking for permeable pavement materials during the design phase, but since it wasn’t well communicated with the suppliers during the design phase, we ran into problems during the construction phase, and they couldn’t find the suitable materials. We thought it was a very commonly available basic material, but actually it wasn’t widely available locally, and the sand used locally did not meet the standard*. **(U10)**

## Conclusions

**Figure 1:**
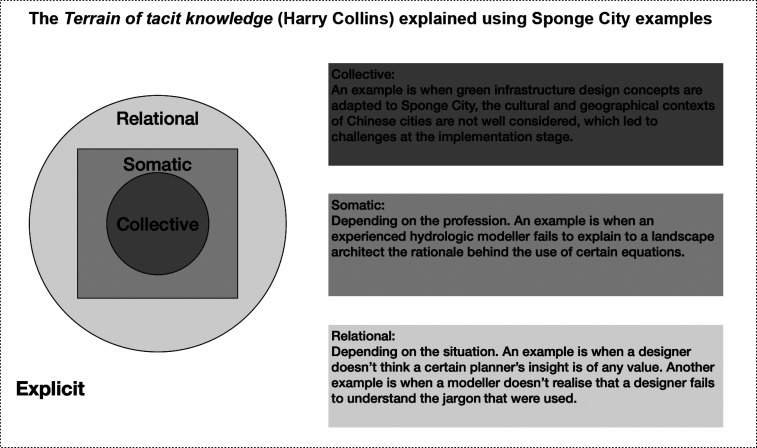
The terrain of tacit knowledge explained using Sponge City examples (adapted from Collins [25]).

Tacit and explicit knowledge are both constructed concepts. There are different takes on where tacit knowledge is located, whether/how/to what extent can it be externalised. Instead of trying to define what tacit knowledge is or is not, what is essential to this particular study is the communication of knowledge that is difficult to transmit from person to person. It is important because the aim of communicating knowledge is to transmit expertise, abilities and competency. In this study, we interviewed actors involved in one to several Sponge City projects sometimes in multiple cities simultaneously or one after the other. They reported difficulties in transferring and communicating knowledge across international borders, government levels, cities, projects, disciplines and even between colleagues. From the examples above we can see that the three types of tacit knowledge are not mutually exclusive, they are nested within one another just as illustrated in Fig. 1. While the CTK cannot be completely explicated, part of it may be explicated if the conditions of communicating the RTK and even STK are met. Cultural factors and the attitudes and behaviours that they are shaping should not be targeted as the root cause of challenges that arise when trying to implement integrated urban water management in China, but it is still necessary to consider the means to bridge as much as possible the communication gaps between water professionals that are contingent upon the limitations of the human bodies, relationships, history, tradition and logistics [20,37,38].

China, as one of the major growing economies, is becoming a protagonist in the adoption and reformulation of sustainable development [39]. The transition of urban water management requires a distinct shift in cognitive, regulative, and normative pillars of institutional practice, and changes to water management are underpinned by political and economic changes [20,34]. Sponge City is China’s attempt to transition towards a more sustainable and resilient urban water management, which ultimately strengthens the public health of the society.

The urban water knowledge network contains a vast amount of existing capacities and competencies to be tapped and shared [40]. However, the discourse of tacit knowledge is infrequently found in the context of urban water management. From the semi-structured interviews conducted with Sponge City actors, examples of RTK, STK and CTK were presented. The terrain of tacit knowledge was used to guide the analysis, which depicts relational as the ‘weakest’, somatic as the ‘weaker’, and collective as the ‘strongest’ tacit knowledge. CTK is the strongest and is tied to the culture and society’s unspoken rules, which is very relevant when we are comparing and adapting across cultural, historical and political boundaries.

Sustainable urban water management faces various challenges due to clashes in understanding, opinions and motivations that present difficulties in inter-disciplinary collaboration and communication. Additional layers of challenges are added because of the cultural and socio-political setting [35,41–43]. This paper categorised the communication difficulties that emerged from the interviews with some Sponge City participants and identified the ‘level of tacitness’ and the conditions of communication they may need. Having fulfilled the goal to distinguish the different types of tacit knowledge and identify examples in the Sponge City case, this will then enable the analysis of the access and mobilisation of different types of tacit knowledge identified. While it is a meaningful exercise to identify the different types of tacit knowledge existing in the Sponge City projects and the programme, we believe the most important is to use this information to further locate the conditions that the communication of each type of tacit knowledge (or each layer of the tacit knowledge) should meet, and their enabling and hindering conditions. This study is therefore the first step in the investigation of the ways water professionals communicate across disciplines and fields in order to achieve sustainable urban water management. The next step is to identify the different types of communication barriers and examine the access and utilisation of knowledge in the Sponge City network by the actors.

## References

[1] Fletcher TD, Shuster W, Hunt WF, Ashley R, Butler D, Arthur S (2015). SUDS, LID, BMPs, WSUD and more – the evolution and application of terminology surrounding urban drainage. Urban Water J.

[2] Mitchell VG (2006). Applying integrated urban water management concepts: a review of Australian experience. Environ Manage.

[3] Wong THF, Brown RR (2009). The water sensitive city: principles for practice. Water Sci Technol.

[4] Cosgrove WJ, Loucks DP (2015). Water management: current and future challenges and research directions. Water Resour Res.

[5] Brown RR, Sharp L, Ashley RM (2006). Implementation impediments to institutionalising the practice of sustainable urban water management. Water Sci Technol.

[6] Grin J, Rotmans J, Schot J (2011). Transitions to Sustainable Development.

[7] MOHURD (2014). 海绵城市建设技术指南-低影响开发雨水系统构建 （试行）(Technical guideline for Sponge City – low impact development stormwater management system construction – provisional).

[8] MOHURD (2018). Assessment standard for Sponge City construction effect.

[9] Yu K, Dihua L, Hong Y, Wei F, Qing Q, Sisi W (2015). ‘Sponge City’: theory and practice. Chengshiguihua.

[10] Ministry of Finance (2016). 关于开展2016年中央财政支持海绵城市建设试点工作的通知 (Notice of Ministry of Finance on providing financial support for Sponge City pilot construction).

[11] Che W, Zhang W (2016). 海绵城市建设若干问题的理性思考 (Sponge City Construction, responding to several questions). Gei Shui Pai Shui.

[12] Geldof GD, Heijden CMG Van Der, Cath AG, Valkman R (2011).

[13] Marlow DR, Moglia M, Cook S, Beale DJ, Land C, Road G (2013). Towards sustainable urban water management: a critical reassessment. Water Res.

[14] Nielsen SB, Jensen MB (2016). Towards sustainable urban water governance in Denmark: collective building of capabilities in local authorities. Int J Innov Sustain Dev.

[15] Wolfe SE (2009). What’s your story? Practitioners’ tacit knowledge and water demand management policies in southern Africa and Canada. Water Policy.

[16] Gourlay S (2002). Tacit knowledge, tacit knowing or behaving?. Oklc.

[17] Loenhoff J, Adloff F, Gerund K, Kaldewey D (2015). Revealing tacit knowledge.

[18] Barron NJ, Kuller M, Yasmin T, Castonguay AC, Copa V, Duncan-Horner E (2017). Towards water sensitive cities in Asia: an interdisciplinary journey. Water Sci Technol.

[19] Bell S (2015). Renegotiating urban water. Prog Plann.

[20] Brown RR, Wong TH, Keath N (2009). Urban water management in cities: historical, current and future regimes. Water Sci Technol.

[21] Dhakal KP, Chevalier LR (2017). Managing urban stormwater for urban sustainability: barriers and policy solutions for green infrastructure application. J Environ Manage.

[22] Qiao X, Kristoffersson A, Randrup TB (2018). Challenges to implementing urban sustainable stormwater management from a governance perspective: a literature review. J Clean Prod.

[23] Polanyi M (1966). The Tacit Dimension.

[24] Polanyi M (1958). Personal Knowledge Towards a Post-Critical Philosophy.

[25] Collins H (2010). Tacit and Explicit Knowledge.

[26] Collins H (2007). Bicycling on the moon: collective tacit knowledge and somatic-limit tacit knowledge. Organ Stud.

[27] Xia J, Zhang YY, Xiong LH, He S, Wang LF, Yu ZB (2017). Opportunities and challenges of the Sponge City construction related to urban water issues in China. Sci China Earth Sci.

[28] Bell S (2018). Urban Water Sustainability: Constructing Infrastructure for Cities and Nature.

[29] Global Water Partnership (2011). Towards integrated urban water management.

[30] Cosier M, Shen D (2009). Urban water management in China. Int J Water Resour Dev.

[31] Lashford C, Rubinato M, Cai Y, Hou J, Abolfathi S, Coupe S (2019). SuDS & sponge cities: a comparative analysis of the implementation of pluvial flood management in the UK and China. Sustain.

[32] Tang YT, Chan FKS, O’Donnell EC, Griffiths J, Lau L, Higgitt DL (2018). Aligning ancient and modern approaches to sustainable urban water management in China: Ningbo as a ‘Blue-Green City’ in the “Sponge City” campaign. J Flood Risk Manag.

[33] Shen D, Liu B (2008). Integrated urban and rural water affairs management reform in China: affecting factors. Phys Chem Earth, Parts A/B/C.

[34] Ball P (2016). The Water Kingdom.

[35] Lo K (2015). How authoritarian is the environmental governance of China?. Environ Sci Policy.

[36] de Jong M, Yu C, Joss S, Wennersten R, Yu L, Zhang X (2015). Eco city development in China: addressing the policy implementation challenge. J Clean Prod.

[37] De Haan FJ, Rogers BC, Frantzeskaki N, Brown RR (2015). Transitions through a lens of urban water. Environ Innov Soc Transitions.

[38] Rogers EM (2003). Diffusion of Innovations.

[39] Olsson J (2009). Sustainable development from below: institutionalising a global idea-complex. Local Environ.

[40] International Water Association (2016). The IWA ‘Principles for Water Wise Cities’ – principles for urban stakeholders to develop a shared vision and liveable cities.

[41] Tan Y, Fang K (2016). Environmental governance in China. J Chinese Gov.

[42] Hou W (1997). Reflections on Chinese traditional ideas of nature. Environ Hist Durh N C.

[43] He G, Lu Y, Mol APJ, Beckers T (2012). Changes and challenges: China’s environmental management in transition. Environ Dev.

